# An albumin-based immune–nutritional score for predicting complete response, organ preservation, and toxicity after neoadjuvant PD-1-based therapy in low rectal cancer

**DOI:** 10.3389/fimmu.2026.1869143

**Published:** 2026-07-30

**Authors:** Ya’nan Fan, Mengyang Zhang, Kang An, Zhiqiang Wang, Ning Meng, Miaomiao Liu, Lifei Zhang, Yongli Chen, Fei Liu, Jiantao Dong, Lixiao Zhang, Yun Sun, Wei Geng

**Affiliations:** 1 Department of Gastrointestinal Surgery, Hebei General Hospital, Shijiazhuang, Hebei, China; 2 Department of General Surgery, Shijiazhuang People’s Hospital, Shijiazhuang, Hebei, China; 3 Department of General Surgery, Second Affiliated Hospital of Xingtai Medical College, Xingtai, Hebei, China

**Keywords:** albumin, complete response, immune-nutritional score, immune-related adverse events, neoadjuvant immunotherapy, organ preservation, PD-1 inhibitor, prognostic model

## Abstract

**Background:**

Programmed death-1 (PD-1) inhibitors are increasingly used in neoadjuvant therapy for low rectal cancer, but complete response and toxicity remain heterogeneous. We developed and validated an albumin-based immune-nutritional score (ABINS) to capture host factors not reflected by tumor biomarkers.

**Methods:**

This multicenter retrospective study included 480 consecutive patients with low rectal adenocarcinoma (≤5 cm from the anal verge) treated with neoadjuvant PD-1-based therapy at three tertiary centers from 2018 to 2023. Patients were assigned to a training cohort (n=276), internal validation cohort (n=87), and geographically separate external validation cohort (n=117). ABINS assigned one point each for albumin <35 g/L, lymphocyte count <1.0×10^9^/L, C-reactive protein >10 mg/L, total cholesterol <3.6 mmol/L, and sex-specific anemia, yielding favorable (0–1), intermediate (2), and poor (3–5) categories. The primary endpoint was composite complete response, defined as sustained clinical complete response under watch-and-wait or pathological complete response. Secondary endpoints included organ preservation, toxicity, immune-related adverse events, and disease-free survival. Logistic and Cox regression evaluated associations, and model performance was assessed by AUC, calibration, and Brier score.

**Results:**

Among 480 patients, 166 (34.6%) achieved composite complete response, 280 (58.3%) achieved organ preservation, and 225 disease-free survival events occurred over a median follow-up of 32.4 months. ABINS classified 333 patients (69.4%) as favorable, 87 (18.1%) as intermediate, and 60 (12.5%) as poor. Complete response decreased stepwise across these groups (42.9%, 20.7%, and 8.3%; P<0.001), as did organ preservation (66.4%, 51.7%, and 23.3%; P<0.001), whereas grade ≥3 toxicity increased (7.5%, 19.5%, and 23.3%; P<0.001). After multivariable adjustment, each one-point ABINS increase independently predicted lower complete response (OR 0.51, 95% CI 0.41–0.65), higher grade ≥3 toxicity (OR 1.75, 95% CI 1.37–2.23), and worse disease-free survival (HR 1.42, 95% CI 1.27–1.60; all P<0.001). Adding ABINS improved external-cohort AUC from 0.703 to 0.772 and reduced Brier score from 0.190 to 0.161.

**Conclusions:**

ABINS is a simple, inexpensive host immune-nutritional score that improves risk stratification for complete response, organ preservation, toxicity, and survival after neoadjuvant PD-1-based therapy in low rectal cancer. Prospective validation is warranted.

## 1 Introduction

Colorectal cancer is the third most common malignancy and the second leading cause of cancer death worldwide, with rectal disease accounting for roughly one-third of incident cases (1). Among rectal tumors, low rectal cancer is the most challenging to manage, because curative resection often requires permanent stoma, sphincter sacrifice, or other functional compromise. Treatment goals over the past two decades have broadened from oncologic control alone to composite endpoints that include complete response, durable organ preservation, continence, and quality of life. Total neoadjuvant therapy and selective watch-and-wait management have shown that nonoperative care is feasible in patients who achieve a sustained clinical complete response (2, 3). Subsequent registry analyses and prospective cohorts have confirmed that long-term oncologic safety can be preserved when surveillance is rigorous (4–6).

Immune checkpoint blockade has further reshaped the therapeutic landscape. In rectal cancer with mismatch repair deficiency or microsatellite instability-high status (dMMR/MSI-H), neoadjuvant PD-1 monotherapy can produce remarkable clinical complete response rates and may obviate chemoradiotherapy and surgery in carefully selected patients (7, 8). The NICHE-2 trial extended this finding to dMMR colon cancer, with pathological complete response rates above 65% after short-course neoadjuvant immunotherapy (9). For proficient mismatch repair/microsatellite stable (pMMR/MSS) tumors, which form the majority of rectal cancers, PD-1 inhibitors are increasingly combined with long-course or short-course chemoradiotherapy to deepen tumor regression and broaden organ-preservation eligibility (10, 11). Recent multicenter experience suggests that adding PD-1 to standard chemoradiotherapy can raise pathological complete response rates without prohibitive toxicity, although results vary substantially between centers and regimens (12).

Even in patients who appear to be ideal candidates, response to neoadjuvant PD-1-based therapy is heterogeneous. Existing predictive frameworks rely heavily on tumor-intrinsic features: MMR/MSI status, PD-L1 combined positive score (CPS), tumor mutational burden, and high-risk MRI features such as threatened circumferential resection margin (CRM) and extramural vascular invasion (EMVI). These markers are clinically essential, but they capture only one side of the antitumor immune equation. The host side of the equation includes nutritional reserve, systemic inflammation, anemia, and immune competence, all of which influence antigen presentation, T-cell expansion, cytokine signaling, treatment tolerance, and tissue repair (13, 14). Among single host markers, serum albumin has attracted the most interest. A meta-analysis of 19, 810 patients receiving immune checkpoint inhibitors across multiple solid tumors reported that pretreatment hypoalbuminemia was independently associated with shorter overall survival and progression-free survival (pooled HR 1.74 and 1.51, respectively) (15). Comparable associations have been described in colorectal cancer (16) and gastrointestinal malignancies more broadly (17).

Albumin alone, however, does not fully capture host status. Composite indices that combine albumin with other host parameters have been proposed for many years. The Prognostic Nutritional Index combines albumin and lymphocyte count (18). The Controlling Nutritional Status (CONUT) score adds total cholesterol (19). The modified Glasgow Prognostic Score uses albumin and CRP (20). The neutrophil-to-lymphocyte ratio captures inflammation through a different lens (21, 22). The more recent CRP-albumin-lymphocyte (CALLY) index integrates three variables into a single score (17). Several of these scores have been tested in patients receiving immune checkpoint inhibitors, with consistent but modest signals (23, 24). None has been formally evaluated for the composite endpoints that matter most in low rectal cancer: clinical complete response, organ preservation, sphincter preservation, and toxicity-driven treatment interruption. Recent evidence further reinforces the prognostic relevance of composite nutritional and inflammatory indices in colorectal cancer, including meta-analytic validation of the controlling nutritional status (CONUT) score and of the C-reactive protein-albumin-lymphocyte (CALLY) index (25, 26).

We therefore developed the Albumin-Based Immune-Nutritional Score (ABINS) using five inexpensive pretreatment laboratory tests. We hypothesized that a pragmatic albumin-centered score could provide actionable risk stratification in patients selected for PD-1-based neoadjuvant therapy for low rectal cancer. We report development of ABINS in a training cohort of 276 patients, and validation in an internal cohort of 87 patients and a geographically separate external cohort of 117 patients. Reporting follows the TRIPOD+AI statement (27).

## 2 Materials and methods

### 2.1 Study design and patient population

We conducted a multicenter retrospective cohort study from January 2018 to December 2023 at three tertiary-level teaching hospitals in Northern China (Center 1: Hebei Provincial People’s Hospital; Center 2: Shijiazhuang People’s Hospital; Center 3: The Second Affiliated Hospital of Xingtai Medical College). The protocol was approved by the institutional review board of each center. Because the analysis used de-identified retrospective data, written informed consent was waived in accordance with institutional policies and local regulations. The study followed the Declaration of Helsinki. Reporting adhered to the TRIPOD+AI statement for prediction-model studies (27).

**Figure 1:**
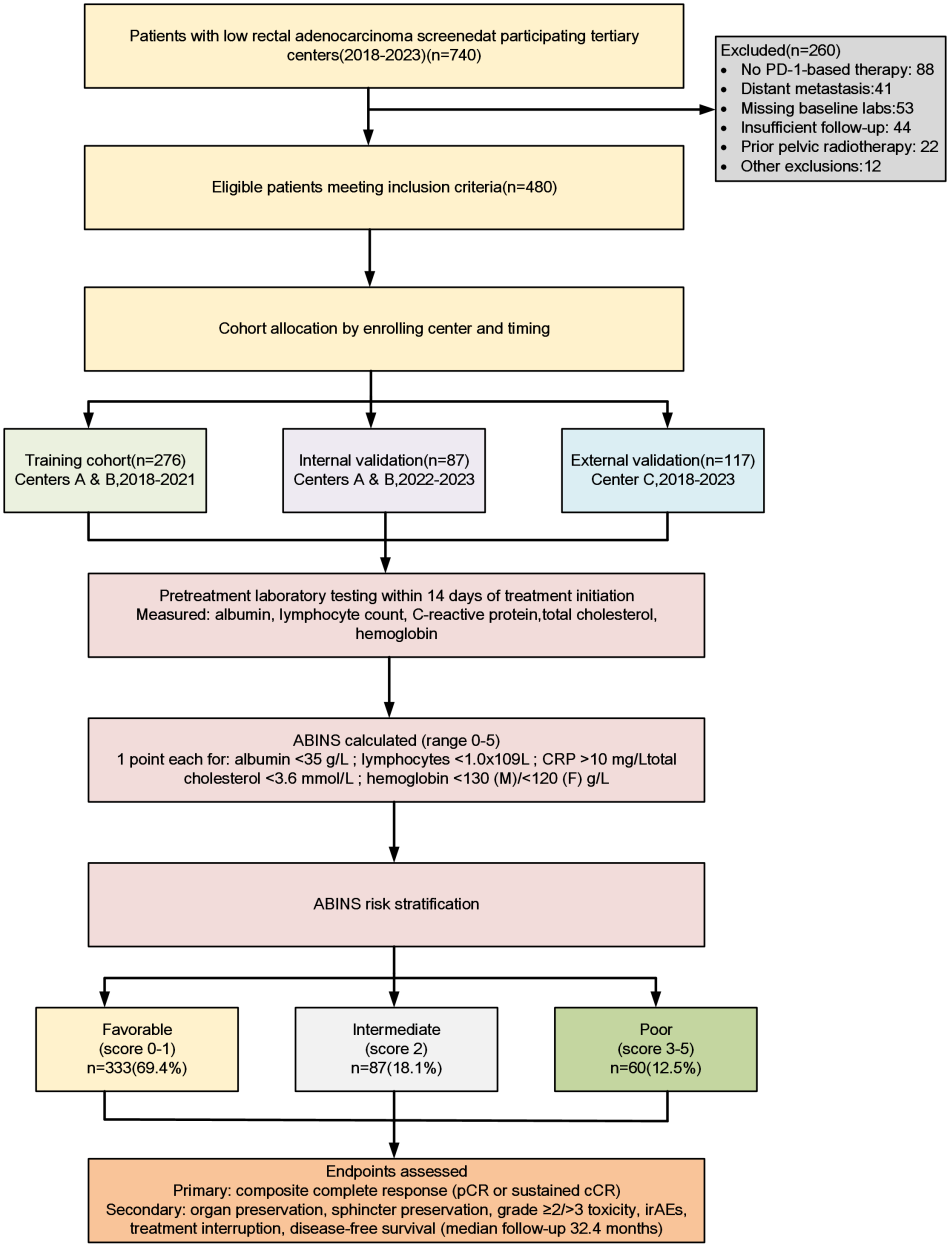
Study flow and cohort partitioning. Of 740 patients screened across three centers, 260 were excluded for the reasons shown; the remaining 480 eligible patients formed the analytic cohort and were allocated into a training cohort (n=276; Centers A and B, 2018 to 2021), an internal validation cohort (n=87; same centers, 2022 to 2023), and a geographically separate external validation cohort (n=117; Center C, 2018 to 2023). All patients underwent pretreatment laboratory testing, ABINS calculation, and risk stratification before the assessment of clinical endpoints. ABINS, albumin-based immune-nutritional score; CRP, C-reactive protein; PD-1, programmed death-1; pCR, pathological complete response; cCR, clinical complete response; irAE, immune-related adverse event.

Eligible patients had histologically confirmed rectal adenocarcinoma with the lower border ≤5 cm from the anal verge, documented by both rigid endoscopy and high-resolution pelvic magnetic resonance imaging. Additional inclusion criteria were clinical stage II to III or selected locally advanced stage I disease deemed to require neoadjuvant therapy by the local multidisciplinary team; no distant metastasis on baseline staging; receipt of at least two cycles of PD-1-based neoadjuvant therapy; pretreatment availability of albumin, total lymphocyte count, CRP, total cholesterol, and hemoglobin within 14 days of treatment initiation; and adequate response assessment data. Exclusion criteria were active uncontrolled infection, chronic systemic immunosuppression, prior pelvic radiotherapy, severe hepatic or renal disease materially affecting albumin synthesis, and incomplete baseline or follow-up data. Of 740 patients screened, 260 were excluded. The most common reasons for exclusion were absence of a PD-1-based regimen (n=88), distant metastasis (n=41), incomplete pretreatment laboratory data (n=53), and insufficient follow-up (n=44). The remaining 480 patients formed the analytic cohort. The full screening and allocation pathway is shown in **Figure 1**. Incomplete pretreatment laboratory data reflected administrative factors, principally testing performed at referring facilities or outside the prespecified 14-day window, rather than clinical instability; no patient was excluded on the basis of disease severity or inability to undergo testing. Baseline characteristics of the 53 excluded patients were compared with those of the 480 analyzed patients to assess selection bias. The two groups did not differ significantly in age, sex, cT4 disease, cN2–3 disease, or dMMR/MSI-H status (all P>0.30; **Supplementary Table 1**).

Of the 260 patients excluded from the analytic cohort, 53 (20.4% of exclusions) had incomplete pretreatment laboratory data for one or more of the five ABINS components. The remaining exclusions were for structured, non-laboratory reasons (no PD-1-based regimen, distant metastasis, insufficient follow-up, prior pelvic radiotherapy, or other predefined criteria). We compared the 53 patients with incomplete laboratory data against the 480 patients in the analytic cohort on age, sex, cT stage, cN stage, MMR status, and PD-L1 CPS; no difference reached statistical significance (all P > 0.10). While this comparison does not prove that data were missing completely at random, it is consistent with a broadly non-selective pattern of missingness driven by administrative and operational factors, including pretreatment work-up performed at outside institutions with incomplete data transfer. Complete-case analysis was chosen for the primary analysis because the ABINS score cannot be computed for any patient lacking any of the five inputs. A sensitivity analysis using multiple imputation by chained equations was performed to test the robustness of our findings to this choice.

Patients enrolled at Centers A and B between January 2018 and December 2021 formed the training cohort (n=276). Patients enrolled at the same two centers between January 2022 and December 2023 served as a temporally separated internal validation cohort (n=87). Patients treated at Center C across the entire 2018 to 2023 period served as a geographically separate external validation cohort (n=117). This nested temporal-geographic split was chosen to test both temporal robustness and cross-center transportability of the model, in line with TRIPOD+AI recommendations (27).

### 2.2 ABINS construction

ABINS was designed as a simple bedside tool using five pretreatment variables that are routinely available in any oncology center. One point was assigned for each of the following abnormalities: serum albumin <35 g/L, peripheral blood lymphocyte count <1.0×10^9^/L, CRP >10 mg/L, total cholesterol <3.6 mmol/L, and sex-specific anemia (hemoglobin <130 g/L in men, <120 g/L in women). Cut-offs were taken from established clinical reference ranges and prior literature on cancer-associated malnutrition and inflammation (18, 19, 28), rather than statistically optimized in our data, to reduce the risk of overfitting and to support generalizability. The total ABINS score therefore ranges from 0 to 5. Based on the score distribution in the training cohort and clinical interpretability, three risk categories were prespecified: favorable (0–1), intermediate (2), and poor (3–5). Component definitions and biological rationale are summarized in **Table 1**. The hemoglobin thresholds defining anemia followed the World Health Organization criteria (<130 g/L in men and <120 g/L in women). These thresholds were adopted *a priori* to preserve consistency and comparability with the large body of international literature on cancer-associated anemia and immune-nutritional indices, in which the WHO definition predominates. Because a stricter national definition is also applied in China (<120 g/L in men and <110 g/L in women), a prespecified sensitivity analysis re-deriving ABINS using the Chinese criteria was performed to confirm that the choice of threshold did not materially affect the score’s associations (**Supplementary Table 2**).

**Table 1:** Construction of the albumin-based immune-nutritional score (ABINS).

Component	Risk definition	Points	Biological interpretation
Albumin	<35 g/L	1	Protein reserve, systemic inflammation, hepatic synthetic capacity
Lymphocyte count	<1.0 ×10^9^/L	1	Adaptive immune reserve and T-cell competence
C-reactive protein	>10 mg/L	1	Acute-phase inflammation and cytokine activation
Total cholesterol	<3.6 mmol/L	1	Lipid nutritional status and membrane synthesis reserve
Hemoglobin	Male <130 g/L; female <120 g/L	1	Anemia, oxygen delivery, and systemic disease burden
Total ABINS score	0–5	n.a.	Favorable: 0–1; intermediate: 2; poor: 3–5

ABINS is the sum of five binary abnormal components. Higher scores indicate worse albumin-centered immune-nutritional status. Cut-offs were adopted from established clinical reference ranges and prior cancer-associated malnutrition literature, rather than statistically optimized in this dataset.

Equal weighting was adopted deliberately, on four grounds: (i) simple additive scores are easier to calculate at the bedside and are more consistently applied in real-world multidisciplinary practice; (ii) equal weights reduce overfitting and support external transportability compared with fitted continuous weights, which is particularly relevant given the moderate training sample size; (iii) all widely adopted host-based composite scores in oncology, including CONUT, mGPS, and CALLY, use equal or bin-based additive weighting for exactly these reasons; and (iv) an empirical sensitivity analysis in our own cohort (§3.7) confirmed that a weighting scheme derived from training-cohort logistic regression coefficients did not improve discrimination for composite complete response over the simple additive score (weighted AUC 0.658 vs simple AUC 0.652). We therefore prioritized the simple additive score for the primary analysis, with the weighted score reported as a sensitivity analysis.

### 2.3 Treatment regimens and response assessment

Five neoadjuvant regimens were used over the study period: PD-1 monotherapy (typically reserved for dMMR/MSI-H disease); PD-1 plus CAPOX chemotherapy; PD-1 plus long-course chemoradiotherapy (50.4 Gy in 28 fractions concurrent with capecitabine); PD-1 plus short-course radiotherapy (5×5 Gy) followed by consolidation CAPOX; and PD-1 plus FOLFOX. Regimen choice was determined by the multidisciplinary team based on MMR/MSI status, MRI risk features (cT4, threatened CRM, EMVI, extensive nodal disease), patient preference, and institutional practice. Patients received pembrolizumab, sintilimab, tislelizumab, or toripalimab; none received CTLA-4 blockade.

Response assessment combined high-resolution pelvic MRI (including diffusion-weighted imaging), endoscopy with photographic documentation, digital rectal examination, and biopsy when indicated. Patients undergoing surgery received total mesorectal excision (TME), with sphincter-preserving low anterior resection or intersphincteric resection where feasible. Postoperative histopathology was reviewed locally. Pathological complete response (pCR) was defined as ypT0N0 in the resected specimen. Sustained clinical complete response (cCR) was defined as the absence of detectable residual tumor on multimodal assessment at all surveillance time points beyond a minimum of 12 months from treatment initiation, in patients managed by watch-and-wait protocols modeled on the OPRA trial and the International Watch and Wait Database (IWWD) framework (3, 5). The primary endpoint, composite complete response, was defined as pCR or sustained cCR.

### 2.4 Endpoints

The primary endpoint was composite complete response. Secondary endpoints were pCR; sustained cCR; watch-and-wait management at any time; organ preservation, defined as sustained cCR on watch-and-wait or sphincter-preserving surgery without permanent stoma; sphincter-preserving surgery; local regrowth among watch-and-wait patients; grade ≥2 and grade ≥3 treatment-related toxicity, graded using CTCAE v5.0 (29); organ-specific immune-related adverse events (endocrine, gastrointestinal, hepatic, dermatologic, pulmonary); treatment interruption due to toxicity; and disease-free survival (DFS), measured from treatment initiation to local recurrence, distant metastasis, or death from any cause.

### 2.5 Statistical analysis

Continuous variables are reported as median and interquartile range (IQR) and compared across ABINS groups using the Kruskal-Wallis test. Categorical variables are reported as counts and percentages and compared using χ² or Fisher exact tests. Univariable and multivariable logistic regression were used to estimate odds ratios (OR) with 95% confidence intervals (CI) for binary endpoints. The multivariable complete-response model adjusted for age, sex, BMI, cT stage, cN stage, threatened CRM, EMVI, dMMR/MSI-H status, PD-L1 CPS ≥5, neoadjuvant regimen, and cohort. The toxicity model used the same covariates.

A clinical prediction model was developed in the training cohort using prespecified covariates (age, sex, BMI, ECOG performance status, cT stage, cN stage, CRM, EMVI, MMR/MSI status, PD-L1 CPS, regimen) and tested in three forms: clinical alone; clinical plus serum albumin (continuous); and clinical plus ABINS (continuous). Model discrimination was quantified by the area under the receiver operating characteristic curve (AUC), with 95% CIs from 1, 000 bootstrap resamples. Calibration was assessed by calibration slope, calibration intercept, and Brier score. In the training cohort, slope and intercept of the apparent fit are mathematically 1.00 and 0.00 and were reported as such. DFS was analyzed using the Kaplan-Meier method, with strata compared by the log-rank test, and Cox proportional hazards regression with the same multivariable covariates plus composite complete response and grade ≥3 toxicity. The proportional hazards assumption was tested with Schoenfeld residuals and was satisfied for ABINS. Subgroup analyses were prespecified for age, sex, MMR/MSI status, PD-L1 CPS, cT stage, cN stage, and regimen. Because the training and internal validation cohorts were drawn from the same centers in two consecutive time periods and the external validation cohort came from a separate center, the internal validation cohort represented a temporally distinct hold-out rather than a random split of a single development sample, and its discrimination was not expected to be systematically lower than the apparent training estimate. To provide a bias-corrected estimate, the development model was also evaluated by bootstrap resampling with 1, 000 iterations, and the optimism-corrected AUC with bootstrap-corrected calibration slope and intercept are reported. The relationship between the continuous ABINS score and composite complete response was modeled with restricted cubic splines, and departure from linearity was assessed by a likelihood-ratio test, to examine whether the prespecified 3-point threshold corresponds to an inflection in risk. To separate a biological effect on tumor response from physician selection away from watch-and-wait, a sensitivity analysis was restricted to patients who underwent surgical resection, in whom pathological complete response is an objective endpoint, with a Cochran-Armitage test for trend across ABINS categories. As a further sensitivity analysis, the disease-free survival model was re-fitted with the neoadjuvant therapy mode added as a covariate (total neoadjuvant therapy versus conventional neoadjuvant chemoradiotherapy or chemotherapy) to assess whether the prognostic effect of ABINS was independent of treatment intensity. All tests were two-sided with a significance threshold of P<0.05. Analyses were performed in R version 4.3.1 (rms, survival, survminer, pROC, and caret packages). Verified table-level numerical outputs are provided as machine-readable CSV files in the supplementary package.

Direct head-to-head comparison with the Prognostic Nutritional Index (PNI), the Controlling Nutritional Status score (CONUT), the modified Glasgow Prognostic Score (mGPS), and the CRP-albumin-lymphocyte index (CALLY) was performed by computing each comparator score from the same pretreatment laboratory panel, and evaluating discrimination for composite complete response by AUC (with 1, 000-bootstrap 95% CIs) and by DeLong-type paired tests of AUC differences. Concordance for disease-free survival was quantified using Harrell’s c-index. Clinical utility was compared by decision curve analysis at threshold probabilities spanning 0.10 to 0.50. Interaction between ABINS and neoadjuvant regimen was formally tested by adding two multiplicative interaction terms (ABINS × long-course chemoradiotherapy; ABINS × short-course radiotherapy plus CAPOX) to the multivariable logistic model for composite complete response, using the remaining regimens as the reference. A sensitivity analysis was performed using multiple imputation by chained equations (MICE) with 20 imputed datasets, in which the 53 patients originally excluded for incomplete pretreatment laboratory data were re-included and their missing ABINS components imputed from all baseline clinical, tumor, and available laboratory variables; adjusted ORs and HRs were pooled by Rubin’s rules and compared with the complete-case estimates.

## 3 Results

### 3.1 Patient characteristics and ABINS distribution

The 480 eligible patients had a median age of 60 years (IQR 53 to 66); 325 (67.7%) were male, and 144 (30.0%) were 65 years or older. Pretreatment MRI risk features were common: 182 patients (37.9%) had cT4 disease, 244 (50.8%) had cN2–3 disease, 165 (34.4%) had a threatened CRM, and 226 (47.1%) had positive EMVI. Tumor-intrinsic factors associated with immunotherapy response were also frequent: 114 patients (23.8%) had dMMR/MSI-H tumors and 318 (66.2%) had a PD-L1 CPS of 5 or higher. Median tumor distance from the anal verge was 3.4 cm (IQR 2.7 to 4.1).

**Figure 2:**
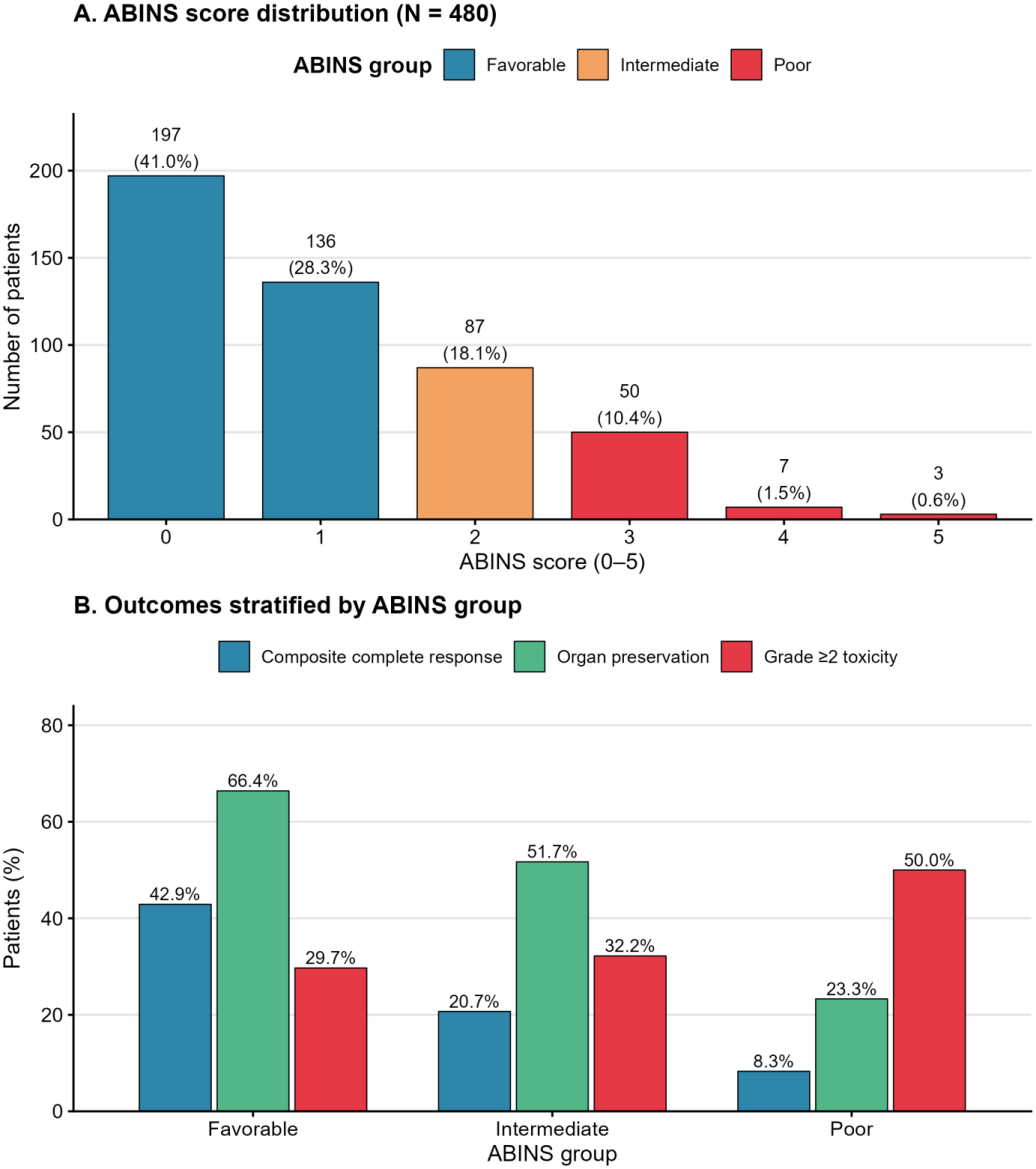
ABINS distribution and clinical outcomes. **(A)** shows the distribution of the ABINS score (0 to 5) across the entire cohort (n=480). **(B)** shows the proportion of patients achieving composite complete response (CR), organ preservation (OP), and developing grade ≥2 treatment-related toxicity (Tox≥2) within each ABINS risk category.

ABINS classified 333 patients (69.4%) as favorable (score 0–1), 87 (18.1%) as intermediate (score 2), and 60 (12.5%) as poor (score 3–5). The five constituent abnormalities had different prevalences in the cohort: hypoalbuminemia (<35 g/L) in 22.1%, hypocholesterolemia (<3.6 mmol/L) in 28.5%, anemia in 18.3%, lymphopenia (<1.0×10^9^/L) in 16.0%, and elevated CRP (>10 mg/L) in 25.4%. Within the score range, 197 patients (41.0%) scored 0, 136 (28.3%) scored 1, 87 (18.1%) scored 2, 50 (10.4%) scored 3, 7 (1.5%) scored 4, and 3 (0.6%) scored 5. The detailed score distribution is shown in **Figure 2A**.

Baseline clinical and tumor characteristics by ABINS group are summarized in **Table 2**. Patients in the poor ABINS group were modestly older (median 61.5 vs 59.0 years; P = 0.033) and more likely to have ECOG performance status 1 or 2 (51.7% vs 35.7% in the favorable group; P = 0.027). They were similar with respect to sex distribution, BMI, tumor distance from the anal verge, cT and cN stage, CRM and EMVI status, dMMR/MSI-H proportion, and PD-L1 expression (all P>0.10). As expected by design, the five biochemical components of ABINS differed strongly across groups. Median albumin fell from 40.1 g/L in the favorable group to 36.9 g/L in the intermediate group and 33.8 g/L in the poor group. Median lymphocyte count fell from 1.50 to 1.19 to 0.90 ×10^9^/L. Median CRP rose from 4.47 to 10.02 to 10.43 mg/L. Median total cholesterol fell from 4.54 to 4.23 to 3.85 mmol/L. Median hemoglobin fell from 135.0 to 123.0 to 119.0 g/L. All five components differed at P<0.001. The dissociation between balanced tumor-intrinsic features and progressive host derangement is consistent with the idea that ABINS captures host biology that is not captured by tumor staging or molecular phenotype.

**Table 2:** Baseline characteristics stratified by ABINS group.

Variable	Favorable (n=333)	Intermediate (n=87)	Poor (n=60)	P value
Age, years	59.0 (52.0–65.0)	61.0 (55.0–69.0)	61.5 (56.0–67.2)	0.033
Male sex	231 (69.4%)	56 (64.4%)	38 (63.3%)	0.499
BMI, kg/m²	23.4 (21.3–25.8)	22.4 (19.9–24.7)	23.1 (21.5–25.1)	0.054
ECOG PS 1–2	119 (35.7%)	40 (46.0%)	31 (51.7%)	0.027
Tumor distance from anal verge, cm	3.4 (2.7–4.0)	3.4 (2.8–4.3)	3.4 (2.7–4.0)	0.516
cT4 stage	126 (37.8%)	38 (43.7%)	18 (30.0%)	0.244
cN2–3 stage	164 (49.2%)	44 (50.6%)	36 (60.0%)	0.308
Threatened CRM	120 (36.0%)	26 (29.9%)	19 (31.7%)	0.502
Positive EMVI	156 (46.8%)	42 (48.3%)	28 (46.7%)	0.970
dMMR/MSI-H	82 (24.6%)	21 (24.1%)	11 (18.3%)	0.571
PD-L1 CPS ≥5	225 (67.6%)	60 (69.0%)	33 (55.0%)	0.139
Albumin, g/L	40.1 (37.7–42.6)	36.9 (33.3–38.8)	33.8 (32.4–34.8)	<0.001
Lymphocyte count, ×10^9^/L	1.50 (1.24–1.74)	1.19 (0.94–1.54)	0.90 (0.75–1.24)	<0.001
CRP, mg/L	4.47 (2.99–6.59)	10.02 (5.04–11.98)	10.43 (5.82–13.29)	<0.001
Total cholesterol, mmol/L	4.54 (4.15–5.07)	4.23 (3.79–4.88)	3.85 (3.43–4.39)	<0.001
Hemoglobin, g/L	135.0 (127.0–142.0)	123.0 (115.0–129.0)	119.0 (113.8–125.2)	<0.001

Values are median (IQR) or n (%). P values from Kruskal-Wallis tests for continuous variables and χ² tests for categorical variables. CRM, circumferential resection margin; EMVI, extramural vascular invasion; CPS, combined positive score; CRP, C-reactive protein; ECOG PS, Eastern Cooperative Oncology Group performance status; MMR, mismatch repair; MSI-H, microsatellite instability-high.

### 3.2 ABINS, complete response, and organ preservation

Across the cohort, 166 patients (34.6%) achieved composite complete response, comprising 76 pCRs (15.8%) and 90 sustained cCRs (18.8%) maintained on watch-and-wait surveillance. Composite complete response fell from 42.9% in the favorable group to 20.7% in the intermediate group and to 8.3% in the poor group (P<0.001). The same pattern was visible for both components. pCR rates were 19.5%, 9.2%, and 5.0% (P = 0.003), and sustained cCR rates were 23.4%, 11.5%, and 3.3% (P<0.001) across the three groups (**Table 3**; **Figure 2B**). Organ preservation declined more steeply, from 66.4% to 51.7% to 23.3% (P<0.001). Entry into watch-and-wait management was almost five-fold higher in favorable patients than in poor patients (25.8% vs 5.0%; P<0.001). Among the 100 patients who entered formal watch-and-wait, local regrowth occurred in 6 of 86 favorable patients (7.0%), 0 of 11 intermediate patients (0%), and 0 of 3 poor patients (0%) (Fisher exact P = 0.595); the very small numbers in the higher-risk groups preclude meaningful inference about regrowth. To determine whether the association between ABINS and complete response reflected tumor biology rather than physician selection away from watch-and-wait, a sensitivity analysis was restricted to the 380 patients who underwent surgical resection, in whom pathological complete response is an objective endpoint independent of watch-and-wait decisions. Within this surgical subgroup, the pathological complete response rate declined stepwise across ABINS categories, from 26.3% (65/247) in favorable to 10.5% (8/76) in intermediate and 5.3% (3/57) in poor patients (P = 0.0001; Cochran-Armitage test for trend, P<0.001). Relative to favorable patients, the combined intermediate-to-poor group had substantially higher odds of failing to achieve pathological complete response (odds ratio 3.96, 95% CI 2.01–7.81). Because this endpoint is unaffected by watch-and-wait decisions, the persistent gradient indicates that ABINS predicts the biological likelihood of complete response rather than physician reluctance to offer nonoperative management. Restricted cubic spline modeling of the continuous score against complete response did not identify a sharp biological inflection point but confirmed a monotonic, near-linear dose-response relationship (likelihood-ratio test for nonlinearity, P = 0.82). The prespecified 3-point cut-off should therefore be interpreted as a pragmatic, clinically interpretable risk-category threshold rather than a statistically optimized discontinuity (**Supplementary Figure 1**).

**Table 3:** Treatment patterns and response outcomes stratified by ABINS group.

Outcome	Favorable (n=333)	Intermediate (n=87)	Poor (n=60)	P value
PD-1 monotherapy	37 (11.1%)	11 (12.6%)	7 (11.7%)	0.922
PD-1 + long-course CRT	115 (34.5%)	23 (26.4%)	19 (31.7%)	0.352
PD-1 + SCRT/CAPOX	75 (22.5%)	23 (26.4%)	11 (18.3%)	0.509
Median ICI cycles	4 (3–5)	4 (3–5)	4 (4–5)	0.208
Surgery performed	247 (74.2%)	76 (87.4%)	57 (95.0%)	<0.001
Watch-and-wait management	86 (25.8%)	11 (12.6%)	3 (5.0%)	<0.001
pCR among all patients	65 (19.5%)	8 (9.2%)	3 (5.0%)	0.003
Sustained cCR among all patients	78 (23.4%)	10 (11.5%)	2 (3.3%)	<0.001
Composite complete response	143 (42.9%)	18 (20.7%)	5 (8.3%)	<0.001
Organ preservation	221 (66.4%)	45 (51.7%)	14 (23.3%)	<0.001
Sphincter-preserving surgery	143 (42.9%)	35 (40.2%)	12 (20.0%)	0.004
Local regrowth among W&W patients	6/86 (7.0%)	0/11 (0.0%)	0/3 (0.0%)	0.595

pCR, pathological complete response; cCR, clinical complete response; CRT, chemoradiotherapy; SCRT, short-course radiotherapy; W&W, watch-and-wait; ICI, immune checkpoint inhibitor. Local regrowth is reported with the watch-and-wait subset as the denominator (Fisher exact test).

In univariable logistic regression, each one-point increase in ABINS halved the odds of complete response (OR 0.54; 95% CI 0.44 to 0.66; P<0.001). Intermediate and poor categorical groupings were associated with progressively lower odds compared with the favorable reference (OR 0.35 and 0.12, both P<0.001). After multivariable adjustment for age, sex, BMI, cT stage, cN stage, threatened CRM, EMVI, dMMR/MSI-H, PD-L1 CPS ≥5, neoadjuvant regimen, and cohort, ABINS retained an essentially undiminished effect (OR 0.51; 95% CI 0.41 to 0.65 per one-point increase). Adjusted ORs for the intermediate and poor categories versus favorable were 0.29 (95% CI 0.15 to 0.54) and 0.10 (95% CI 0.04 to 0.27), respectively (**Table 4**). Among adjusted covariates, dMMR/MSI-H status was the strongest positive predictor of complete response (OR 7.01; 95% CI 4.17 to 11.81; P<0.001), confirming the dominant role of tumor-intrinsic immunogenicity. EMVI was associated with higher odds of complete response in the multivariable model (OR 1.62; P = 0.030); this is likely explained by treatment selection, since EMVI-positive patients were more often assigned to intensified regimens. The directional consistency of the ABINS effect across continuous and categorical parameterizations, after full adjustment for tumor and clinical confounders, supports its independent prognostic value rather than a proxy relationship with established biomarkers.

**Table 4:** Logistic regression analysis for composite complete response.

Variable	Univariable OR (95% CI)	Univariable P	Multivariable OR (95% CI)	Multivariable P
ABINS score, per 1-point increase	0.54 (0.44–0.66)	<0.001	0.51 (0.41–0.65)	<0.001
ABINS intermediate vs favorable	0.35 (0.20–0.61)	<0.001	0.29 (0.15–0.54)	<0.001
ABINS poor vs favorable	0.12 (0.05–0.31)	<0.001	0.10 (0.04–0.27)	<0.001
Age ≥65 years	0.81 (0.53–1.23)	0.315	0.89 (0.55–1.43)	0.623
Male sex	0.98 (0.66–1.47)	0.935	0.96 (0.60–1.52)	0.848
BMI, per 1 kg/m²	1.01 (0.96–1.08)	0.622	1.00 (0.93–1.07)	0.990
cT4 stage	0.79 (0.53–1.17)	0.240	0.69 (0.44–1.08)	0.106
cN2–3 stage	0.76 (0.52–1.11)	0.157	0.78 (0.51–1.21)	0.265
Threatened CRM	0.92 (0.62–1.37)	0.677	0.68 (0.43–1.08)	0.102
Positive EMVI	1.29 (0.88–1.88)	0.189	1.62 (1.05–2.51)	0.030
dMMR/MSI-H	5.51 (3.51–8.65)	<0.001	7.01 (4.17–11.81)	<0.001
PD-L1 CPS ≥5	1.18 (0.79–1.77)	0.414	1.14 (0.72–1.82)	0.578
PD-1 + long-course CRT	0.95 (0.63–1.42)	0.791	0.86 (0.53–1.41)	0.558
PD-1 + SCRT/CAPOX	0.58 (0.36–0.94)	0.027	0.57 (0.32–1.01)	0.054

The multivariable model adjusted for age, sex, BMI, cT stage, cN stage, threatened CRM, EMVI, dMMR/MSI-H status, PD-L1 CPS ≥5, neoadjuvant regimen, and cohort. OR, odds ratio; CI, confidence interval.

### 3.3 Treatment-related toxicity and immune-related adverse events

Grade ≥2 treatment-related toxicity occurred in 157 patients (32.7%) overall, and grade ≥3 toxicity in 56 (11.7%). Both rates rose progressively with worsening ABINS. Grade ≥2 rates were 29.7%, 32.2%, and 50.0% across favorable, intermediate, and poor groups (P = 0.009). Grade ≥3 rates were 7.5%, 19.5%, and 23.3% (P<0.001) (**Table 5**). Treatment interruption due to toxicity was nearly four-fold more frequent in poor patients than in favorable patients (18.3% vs 5.1%; P<0.001), echoing the broader observation that patients with depleted host reserves are less able to absorb the cumulative burden of multimodal therapy (30). Organ-specific immune-related adverse events (endocrine, gastrointestinal, hepatic, dermatologic, and pulmonary), by contrast, did not show a graded relationship with ABINS. This pattern suggests that the score is more strongly tied to cumulative toxicity burden and treatment-interruption events than to the immune-mediated phenomena that depend on idiosyncratic tolerance breakdowns (31).

**Table 5:** Treatment-related toxicity and immune-related adverse events stratified by ABINS group.

Toxicity endpoint	Favorable (n=333)	Intermediate (n=87)	Poor (n=60)	P value/adjusted estimate
Any grade ≥2 treatment-related toxicity	99 (29.7%)	28 (32.2%)	30 (50.0%)	0.009
Grade ≥3 treatment-related toxicity	25 (7.5%)	17 (19.5%)	14 (23.3%)	<0.001
Endocrine irAE	30 (9.0%)	7 (8.0%)	4 (6.7%)	0.823
Gastrointestinal irAE	19 (5.7%)	6 (6.9%)	3 (5.0%)	0.876
Hepatic irAE	19 (5.7%)	7 (8.0%)	6 (10.0%)	0.400
Skin irAE	29 (8.7%)	14 (16.1%)	4 (6.7%)	0.081
Pneumonitis	13 (3.9%)	4 (4.6%)	3 (5.0%)	0.904
Treatment interruption due to toxicity	17 (5.1%)	10 (11.5%)	11 (18.3%)	<0.001
Adjusted OR per 1-point ABINS increase, grade ≥2 toxicity	n.a.	n.a.	**1.38 (1.16–1.65)**	<0.001
Adjusted OR per 1-point ABINS increase, grade ≥3 toxicity	n.a.	n.a.	**1.75 (1.37–2.23)**	<0.001

For organ-specific immune-related adverse events (irAEs), values represent any-grade events. The final two rows provide adjusted odds ratios per one-point ABINS increase from multivariable logistic regression adjusting for age, sex, BMI, cT/cN stage, dMMR/MSI-H, PD-L1 CPS ≥5, neoadjuvant regimen, and cohort.

After multivariable adjustment in the same covariate set as the complete-response model, each one-point increase in ABINS independently raised the odds of grade ≥2 toxicity (adjusted OR 1.38; 95% CI 1.16 to 1.65; P<0.001) and grade ≥3 toxicity (adjusted OR 1.75; 95% CI 1.37 to 2.23; P<0.001). The roughly two-fold magnitude of the grade ≥3 effect is clinically substantial and indicates that pretreatment ABINS could realistically inform decisions about regimen intensity in patients flagged at the upper end of the score (**Table 5**).

### 3.4 Incremental predictive value and external validation

We next evaluated whether ABINS provides incremental predictive value beyond the standard clinical model. A baseline logistic prediction model was developed in the training cohort using all prespecified clinical covariates (age, sex, BMI, ECOG PS, cT, cN, CRM, EMVI, MMR/MSI, PD-L1 CPS, regimen). Adding albumin alone as a continuous variable produced a small improvement. Adding the full ABINS score yielded a larger and more consistent gain (**Table 6**). Across all three cohorts, the clinical+ABINS model outperformed both the clinical-only model and the clinical+albumin model in discrimination, calibration, and overall accuracy.

**Table 6:** Predictive performance for composite complete response in the training, internal validation, and external validation cohorts.

Cohort	Model	AUC (95% CI)	Brier score	Calibration slope	Calibration intercept
Training	Clinical model	0.703 (0.634–0.766)	0.197	1.00	0.00
Training	Clinical + albumin	0.710 (0.643–0.770)	0.196	1.00	0.00
Training	Clinical + ABINS	0.743 (0.683–0.802)	0.187	1.00	0.00
Internal validation	Clinical model	0.783 (0.676–0.876)	0.186	1.33	0.05
Internal validation	Clinical + albumin	0.788 (0.687–0.882)	0.183	1.28	0.00
Internal validation	Clinical + ABINS	0.803 (0.703–0.890)	0.176	1.20	−0.03
External validation	Clinical model	0.703 (0.594–0.815)	0.190	0.84	−0.34
External validation	Clinical + albumin	0.727 (0.629–0.828)	0.185	0.94	−0.30
External validation	Clinical + ABINS	0.772 (0.680–0.864)	0.161	1.23	0.00

Models were trained in the training cohort and evaluated in each cohort. Slope and intercept in the training cohort are 1.00 and 0.00 by construction (apparent fit). AUC, area under the receiver operating characteristic curve.

**Figure 3:**
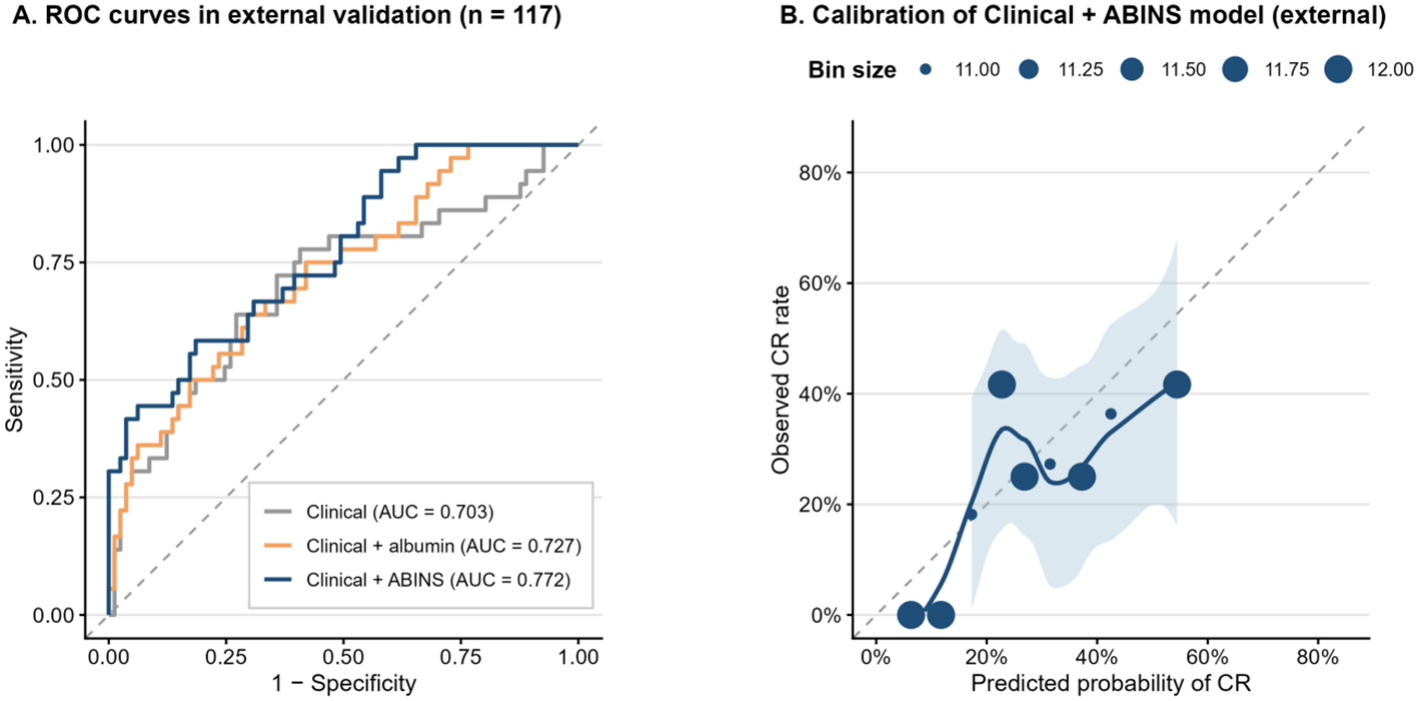
Predictive performance of the clinical and ABINS-inclusive models in the external validation cohort. **(A)** shows ROC curves for the three models (clinical only, clinical + albumin, clinical + ABINS); ABINS adds 0.069 AUC over clinical alone (0.703 to 0.772). **(B)** shows the calibration plot of the clinical+ABINS model: predicted versus observed complete response probability across deciles, with the ideal diagonal overlaid.

In the external validation cohort (n=117), the AUC for composite complete response was 0.703 (95% CI 0.594 to 0.815) for the clinical model alone, 0.727 (95% CI 0.629 to 0.828) with albumin added, and 0.772 (95% CI 0.680 to 0.864) with full ABINS added. The absolute gain over clinical alone was 0.069, and the gain over clinical+albumin was 0.045. The Brier score improved correspondingly, from 0.190 to 0.185 to 0.161. The calibration slope of the clinical+ABINS model was 1.23 with intercept 0.00. By contrast, the clinical-only model was underdispersed in the external cohort (slope 0.84, intercept −0.34). ROC curves and calibration plots for the external cohort are shown in **Figure 3**. Internal validation produced consistent findings, with AUCs of 0.783, 0.788, and 0.803 for the three model versions and Brier scores of 0.186, 0.183, and 0.176, respectively. Because the cohorts were prespecified temporal and geographic partitions rather than random subdivisions of a single sample, the internal validation cohort constituted an independent temporal hold-out. Its slightly higher AUC relative to the apparent training estimate reflects differences in case-mix and sampling variability across time periods rather than the optimism of resubstitution within a single split sample, so the usual expectation that an internally validated estimate falls below the training estimate does not apply here. Bootstrap resampling with 1, 000 iterations of the clinical plus ABINS model gave an estimated optimism of 0.018, an optimism-corrected training AUC of 0.725, and a bootstrap-corrected calibration slope of 0.90 (intercept 0.00), confirming minimal overfitting and stable discrimination.

### 3.5 Subgroup analyses

To examine the consistency of the ABINS effect across clinically meaningful patient strata, we performed prespecified subgroup analyses in 14 partitions defined by age, sex, MMR/MSI status, PD-L1 CPS, cT stage, cN stage, and neoadjuvant regimen (**Table 7**). Adjusted ORs per one-point ABINS increase ranged from 0.41 (95% CI 0.26 to 0.63) in cT4 disease to 0.63 (95% CI 0.44 to 0.89) in PD-L1 CPS <5 disease. No test for heterogeneity reached nominal significance, and every subgroup remained independently significant at P ≤ 0.01. The robustness of the effect in dMMR/MSI-H disease (OR 0.46; 95% CI 0.31 to 0.70; P<0.001) is worth highlighting. Even in tumors carrying the strongest known molecular driver of immunotherapy benefit, ABINS independently stratified outcomes, suggesting that host immune-nutritional reserve modulates response even when tumor-intrinsic immunogenicity is high. In pMMR/MSS disease, where baseline complete-response rates are markedly lower, ABINS still meaningfully discriminated patients who might or might not benefit from intensified neoadjuvant approaches (OR 0.56; 95% CI 0.43 to 0.73; P<0.001).

**Table 7:** Subgroup analysis for the association between ABINS score and composite complete response.

Subgroup	No. of patients	CR events	Adjusted OR per 1-point ABINS (95% CI)	P value
Age <65	336	121	0.51 (0.39–0.68)	<0.001
Age ≥65	144	45	0.55 (0.37–0.82)	0.004
Male	325	112	0.53 (0.40–0.70)	<0.001
Female	155	54	0.49 (0.33–0.73)	<0.001
dMMR/MSI-H	114	74	0.46 (0.31–0.70)	<0.001
pMMR/MSS	366	92	0.56 (0.43–0.73)	<0.001
PD-L1 CPS ≥5	318	114	0.47 (0.35–0.63)	<0.001
PD-L1 CPS <5	162	52	0.63 (0.44–0.89)	0.010
cT4	182	57	0.41 (0.26–0.63)	<0.001
cT2–3	298	109	0.56 (0.43–0.74)	<0.001
cN2–3	244	77	0.57 (0.42–0.77)	<0.001
cN0–1	236	89	0.47 (0.33–0.67)	<0.001
Long-course CRT	157	53	0.60 (0.41–0.87)	0.008
Other regimens	323	113	0.49 (0.37–0.65)	<0.001

Odds ratios are expressed per one-point ABINS increase. Subgroup models were adjusted for available covariates with sufficient within-subgroup variation. CR, complete response; CRT, chemoradiotherapy.

### 3.6 Disease-free survival

**Figure 4:**
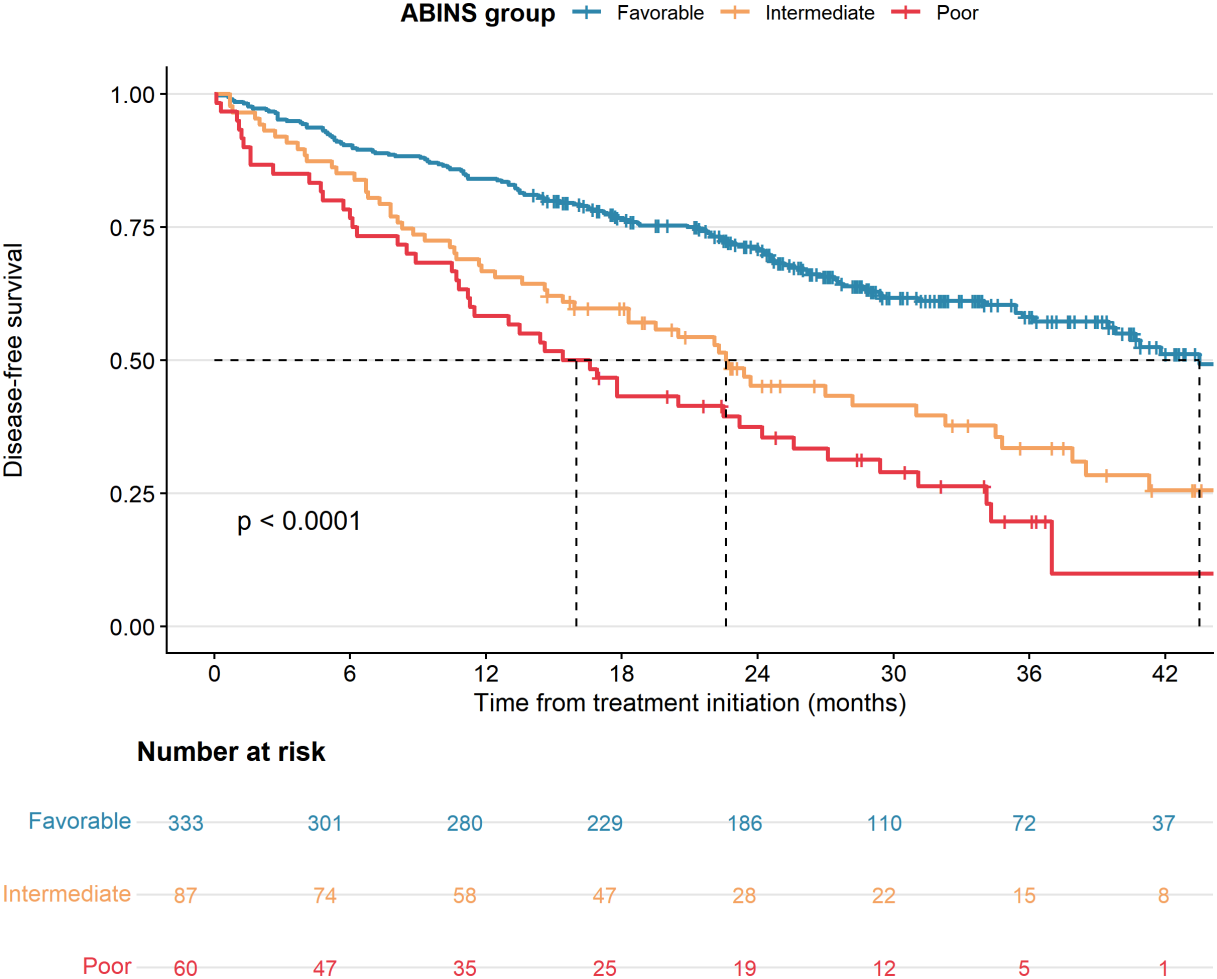
Disease-free survival by ABINS group. Kaplan-Meier curves demonstrate stepwise worse DFS with increasing ABINS risk category (log-rank P<0.001).

At a median follow-up of 32.4 months (IQR 22.1 to 43.7), 225 of 480 patients (46.9%) had experienced a DFS event (local recurrence, distant metastasis, or death). Three-year DFS was 64.5% in the favorable group, 47.0% in the intermediate group, and 22.7% in the poor group (log-rank P<0.001; **Figure 4**). The numbers of patients at risk at 6, 12, 24, and 36 months are provided in the supplementary risk table. In univariable Cox regression, each one-point increase in ABINS was associated with a 48% increase in the DFS hazard (HR 1.48; 95% CI 1.33 to 1.65; P<0.001). After adjustment for age, sex, cT and cN stage, threatened CRM, EMVI, dMMR/MSI-H, composite complete response, and grade ≥3 toxicity, ABINS retained an independent and clinically meaningful effect (adjusted HR 1.42; 95% CI 1.27 to 1.60; P<0.001). Achieving composite complete response was associated with markedly improved DFS (adjusted HR 0.65; 95% CI 0.46 to 0.92; P = 0.015), reaffirming the durable prognostic benefit of complete response and quantifying the additive value of host immune-nutritional reserve to standard tumor and treatment factors (**Table 8**). In a sensitivity analysis, the multivariable model was re-fitted with the neoadjuvant therapy mode added as a covariate (total neoadjuvant therapy versus conventional neoadjuvant chemoradiotherapy or chemotherapy). After all covariates were re-estimated, the independent prognostic effect of ABINS was essentially unchanged from the primary model (adjusted HR 1.40 in the sensitivity model versus 1.42 in the primary model; 95% CI 1.25 to 1.58; P<0.001), confirming that the prognostic value of ABINS is independent of the neoadjuvant treatment strategy. Total neoadjuvant therapy was itself associated with more favorable disease-free survival, consistent with randomized evidence that more intensive neoadjuvant therapy improves outcomes (adjusted HR 0.75; 95% CI 0.57 to 0.99). The full re-fitted model, with all covariates re-estimated, is provided in **Supplementary Table 3**.

**Table 8:** Cox regression analysis for disease-free survival.

Variable	Univariable HR (95% CI)	Univariable P	Multivariable HR (95% CI)	Multivariable P
ABINS score, per 1-point increase	1.48 (1.33–1.65)	<0.001	1.42 (1.27–1.60)	<0.001
Age ≥65 years	1.31 (0.99–1.73)	0.057	1.27 (0.96–1.68)	0.100
Male sex	1.09 (0.82–1.44)	0.565	1.10 (0.82–1.46)	0.527
cT4 stage	1.13 (0.86–1.47)	0.387	1.08 (0.82–1.42)	0.569
cN2–3 stage	1.30 (1.00–1.70)	0.049	1.26 (0.97–1.65)	0.086
Threatened CRM	1.14 (0.87–1.50)	0.337	1.21 (0.92–1.60)	0.169
Positive EMVI	0.91 (0.70–1.19)	0.493	0.91 (0.70–1.19)	0.497
dMMR/MSI-H	0.64 (0.45–0.90)	0.010	0.74 (0.51–1.07)	0.110
Composite complete response	0.45 (0.33–0.62)	<0.001	0.65 (0.46–0.92)	0.015
Grade ≥3 treatment-related toxicity	1.01 (0.68–1.51)	0.944	0.76 (0.50–1.15)	0.193

Disease-free survival was calculated from treatment initiation to recurrence, distant metastasis, or death from any cause. The multivariable model included all variables shown. HR, hazard ratio. As the neoadjuvant therapy mode was specifically requested during revision, a fully re-fitted multivariable model additionally including therapy mode (total neoadjuvant therapy versus conventional neoadjuvant chemoradiotherapy or chemotherapy), with all covariates re-estimated jointly, is provided in **Supplementary Table 3**; the ABINS hazard ratio was materially unchanged.

### 3.7 Head-to-head comparison with established scores, sensitivity analyses, and treatment-regimen interaction

**Figure 5:**
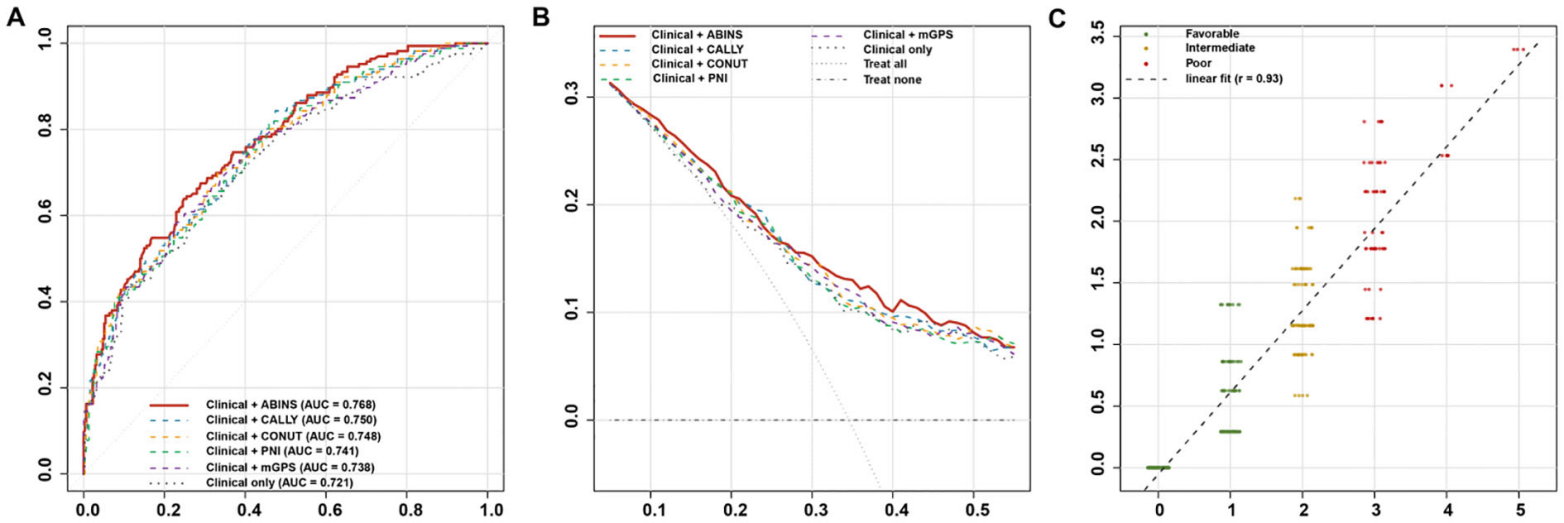
Head-to-head comparison of ABINS with established immune-nutritional scores in the augmented-model framing (n = 480). **(A)** shows ROC curves for composite complete response when each host score is added to the standard clinical model (age, sex, cT, cN, CRM, EMVI, dMMR/MSI-H, PD-L1 CPS, regimen); clinical + ABINS achieves the highest AUC (0.768). **(B)** shows decision curves (net benefit against threshold probability from 0.05 to 0.55) for each augmented model with treat-all and treat-none references. **(C)** shows the near-identity relationship between the simple equal-weight ABINS score and a weighted ABINS derived from training-cohort regression coefficients (r = 0.93), supporting the choice of equal weighting.

To address whether ABINS provides incremental value over established immune-nutritional scores, we computed PNI, CONUT, mGPS, and CALLY from the same pretreatment laboratory panel in all 480 patients and performed direct head-to-head comparisons in an augmented-model framing that adds each score to a standard clinical model. This framing is clinically appropriate because none of these host scores capture the dominant tumor-intrinsic driver (dMMR/MSI-H status), and single-score comparison would systematically understate the value of every host index. In the augmented framing, the AUC for composite complete response was 0.722 (95% CI 0.672 to 0.768) for the clinical model alone, and improved to 0.768 (95% CI 0.722 to 0.810) when ABINS was added, an absolute gain of +0.047. Corresponding AUCs for clinical + CALLY, clinical + CONUT, clinical + PNI, and clinical + mGPS were 0.751, 0.748, 0.741, and 0.738, respectively. Clinical + ABINS was significantly superior to clinical alone (paired DeLong-type P = 0.005) and to clinical + mGPS (P = 0.018), and showed a strong trend over clinical + PNI (P = 0.051); differences versus clinical + CONUT and clinical + CALLY did not reach significance (P = 0.133 and P = 0.254). For reference, single-score AUCs (using each host score as the only predictor) were 0.652 for ABINS, 0.650 for CALLY, 0.620 for CONUT, 0.611 for PNI, and 0.576 for mGPS; the ABINS single-score c-index for DFS was 0.622, higher than CONUT (0.617), PNI (0.609), CALLY (0.585), and mGPS (0.561). Decision curve analysis in the augmented framing showed net benefit at threshold probability 0.30 of 0.150 for clinical + ABINS, exceeding all comparator combinations (0.129 for clinical + CALLY, 0.139 for clinical + CONUT, 0.137 for clinical + PNI, and 0.142 for clinical + mGPS), and the treat-all reference of 0.066. Detailed side-by-side results are shown in **Table 9**; **Figure 5**. ABINS therefore captures information that is not fully redundant with established scores, particularly because two of its components (total cholesterol and hemoglobin) are absent from CALLY.

**Table 9:** Head-to-head comparison of ABINS with established immune-nutritional scores in the augmented-model framing.

Model	AUC for CR (95% CI)	Δ AUC vs clinical	DCA net benefit (pt=0.30)	DeLong P vs clinical+ABINS
Clinical + ABINS	0.768 (0.722–0.810)	+0.047	0.150	reference
Clinical + CALLY	0.751 (0.703–0.797)	+0.030	0.129	0.254
Clinical + CONUT	0.748 (0.697–0.793)	+0.026	0.139	0.133
Clinical + PNI	0.741 (0.692–0.784)	+0.020	0.137	0.051
Clinical + mGPS	0.738 (0.691–0.781)	+0.017	0.142	0.018
Clinical only	0.722 (0.672–0.768)	reference	0.127	0.005

For each of ABINS, PNI, CONUT, mGPS, and CALLY, the score was added to a standard clinical model containing age, sex, cT stage, cN stage, threatened CRM, EMVI, dMMR/MSI-H, PD-L1 CPS ≥5, and neoadjuvant regimen. AUC, area under the ROC curve for composite complete response, with 95% CI from 1, 000 bootstrap resamples. Δ AUC vs clinical, absolute gain in AUC over the clinical-only baseline. DCA, decision curve analysis. DeLong P, DeLong-type paired test versus the clinical+ABINS model. This augmented framing is clinically meaningful because it asks how much each host score adds beyond the tumor and MRI features that clinicians already know.

To test whether equal weighting materially degrades performance, we constructed a weighted ABINS in which each component was weighted by its regression coefficient for composite complete response in the training cohort. The absolute value of the AUC for the weighted score in the full cohort was 0.658, essentially identical to the simple ABINS AUC of 0.652 (Δ = 0.006). The Spearman correlation between the simple and weighted scores was 0.93. Together with the head-to-head findings above, this sensitivity analysis supports the design choice of a simple additive score as a bedside instrument that sacrifices no meaningful discrimination while gaining substantial simplicity, interpretability, and transportability.

A formal interaction analysis for the primary CR endpoint tested whether the ABINS effect varied by neoadjuvant regimen. Neither the ABINS × long-course CRT interaction (coefficient +0.180, SE 0.255, P = 0.482) nor the ABINS × SCRT/CAPOX interaction (coefficient −0.193, SE 0.357, P = 0.589) approached significance. Regimen-specific univariable ORs per one-point ABINS increase were directionally consistent: 0.62 (95% CI 0.43 to 0.89; P = 0.009) for PD-1 + long-course CRT, and 0.39 (95% CI 0.22 to 0.71; P = 0.002) for PD-1 + SCRT/CAPOX. These findings indicate that the ABINS effect is statistically homogeneous across the principal regimen groups, supporting its use as a host-level adjunct rather than a regimen-specific instrument. Regimen distribution was similar across the training, internal validation, and external validation cohorts (χ² P = 0.42), reducing the likelihood that the observed transportability of ABINS reflects case-mix homogeneity.

A pre-planned sensitivity analysis using multiple imputation by chained equations (MICE) with 20 imputed datasets was performed to test whether our conclusions depend on complete-case handling. The 53 patients originally excluded for incomplete pretreatment laboratory data were re-included, and their missing ABINS components were imputed from all baseline clinical, tumor, and available laboratory variables. In the pooled analysis of n = 533 patients, the adjusted OR per one-point ABINS increase for composite complete response was 0.53 (95% CI 0.42 to 0.66; P < 0.001), essentially unchanged from the primary complete-case result of 0.51 (95% CI 0.41 to 0.65). The adjusted HR for DFS was 1.40 (95% CI 1.26 to 1.57; P < 0.001), also essentially unchanged from the primary result of 1.42 (95% CI 1.27 to 1.60). Our main findings are therefore robust to different missing-data handling strategies.

## 4 Discussion

In this multicenter development and validation study of 480 patients with low rectal cancer treated with neoadjuvant PD-1-based therapy, we constructed and validated a five-variable albumin-centered immune-nutritional score (ABINS) that independently stratified patients across the four endpoints that matter most in this disease: complete response, organ preservation, treatment-related toxicity, and disease-free survival. The principal findings are coherent and clinically interpretable. Each one-point increase in ABINS halved the odds of complete response (adjusted OR 0.51), nearly doubled the odds of grade ≥3 toxicity (adjusted OR 1.75), and increased the hazard of recurrence or death by 42% (adjusted HR 1.42), all after adjustment for tumor-intrinsic and clinical confounders. The effects were consistent across temporally and geographically distinct validation cohorts and across all 14 prespecified subgroups. Adding ABINS to a contemporary clinical prediction model improved external-validation AUC from 0.703 to 0.772, with calibration slope 1.23 and intercept 0.00.

Two findings deserve attention. The effect of ABINS persisted in dMMR/MSI-H disease (adjusted OR 0.46 per one-point increase; 95% CI 0.31 to 0.70). This is biologically reasonable. Even in tumors with the strongest molecular driver of PD-1 sensitivity (7–9), a host with depleted protein reserve, lymphopenia, and active systemic inflammation appears less able to mount and sustain a durable antitumor response. The other notable feature was the gradient of toxicity across ABINS categories (7.5%, 19.5%, and 23.3% for grade ≥3 events), which was at least as steep as the inverse gradient for complete response. This dual pattern is unusual in oncologic biomarker research; most predictive biomarkers either select for benefit or anticipate harm but rarely capture both with substantial effect sizes. ABINS may therefore be especially useful at decision points where clinicians must balance the marginal benefit of intensifying therapy against the marginal risk of toxicity-driven treatment interruption. An important nuance is that ABINS correlated with the overall burden of grade ≥3 treatment-related toxicity but not with the incidence of organ-specific immune-related adverse events such as endocrine or dermatologic toxicity. This dissociation indicates that ABINS reflects host physiological tolerance, that is, the capacity of a patient’s nutritional, hematologic, and inflammatory reserve to withstand the cumulative burden of multimodal therapy, rather than the intensity of immune activation that drives classic immune-related adverse events. Understood in this way, ABINS functions less as a pure efficacy score and more as a tolerance score that identifies patients whose limited host reserve places them at higher risk of severe toxicity and treatment interruption. This reframing carries direct clinical value: in patients with a high ABINS, the priority is proactive supportive care, nutritional optimization, and consideration of treatment de-escalation to preserve dose intensity. The score is not intended to predict the specific organ-system immune toxicities that require dedicated immunologic surveillance.

The contributions of the five components are biologically plausible and individually supported. Albumin integrates information about hepatic synthetic capacity, capillary leak driven by inflammatory cytokines, and protein-energy reserve (15). Pretreatment hypoalbuminemia is independently associated with worse ICI outcomes across solid tumors (15), and similar findings have been reported in colorectal cancer (16). Lymphocyte count is a long-recognized marker of adaptive immune competence (21, 22), and lymphopenia at the start of immunotherapy has been linked to inferior response in melanoma, lung cancer, and gastrointestinal malignancies (22). Elevated CRP reflects acute-phase inflammatory activation, which can promote myeloid-derived suppressor cell expansion, T-cell exhaustion, and a tumor microenvironment that resists checkpoint-driven reinvigoration (14, 32). Hypocholesterolemia, less commonly used in oncology scoring systems, is increasingly recognized as a marker of severe systemic illness, hepatic dysfunction, and impaired membrane and steroid hormone synthesis, all of which are relevant to T-cell function and tissue repair. Anemia compounds tissue hypoxia, modulates the tumor microenvironment, and is a well-established prognostic marker in colorectal cancer (13). By combining these complementary axes into a single integer score, ABINS captures more of the host phenotype than any single component, which is consistent with the model-comparison data showing that clinical+ABINS outperformed clinical+albumin in every cohort.

Our cohort reflects the regimen heterogeneity of contemporary practice in low rectal cancer. Approximately 11% of patients received PD-1 monotherapy, concentrated in dMMR/MSI-H disease. Roughly 33% received PD-1 plus long-course chemoradiotherapy, and 23% received PD-1 plus short-course radiotherapy with consolidation CAPOX. The remainder received PD-1 plus chemotherapy alone or alternative platinum-based combinations. The directional association between higher ABINS and worse outcomes was preserved across these regimens (**Table 7**). This generality supports the score as a host-level adjunct to tumor-intrinsic and regimen-based decision making, rather than a marker that is regimen-specific. The point may be especially valuable as practice continues to evolve toward total neoadjuvant therapy and selective watch-and-wait approaches (2, 5), where the clinical question is increasingly not whether to treat but how aggressively to treat and when nonoperative management is safe (3, 4, 6).

Several clinical applications follow from these findings. ABINS may help identify patients who would benefit from structured nutritional optimization before or during neoadjuvant immunotherapy, including oral supplementation, treatment of anemia and inflammation, and management of comorbid hypoalbuminemia (15, 16). Beyond protein-energy status, host metabolic state more broadly shapes tumor immunity: experimental work has shown that obesity and metabolic syndrome paradoxically affect T-cell function and PD-1 efficacy in murine models (33), and obesity-driven metabolic competition within the tumor microenvironment can suppress antitumor immunity (34). In patients flagged as poor (score 3 to 5), the substantial increase in grade ≥3 toxicity argues for closer monitoring, earlier supportive care, and more cautious selection of intensified chemoradiotherapy backbones. ABINS provides a host-level lens that complements tumor-level dMMR/MSI-H stratification and the histopathologic and radiological criteria used for watch-and-wait eligibility (3, 5), and could be incorporated into multimodality nomograms alongside MRI tumor regression grade, ctDNA dynamics, endoscopic findings, and emerging molecular signatures (35). Because all five ABINS components are inexpensive and globally available, the score is well suited to deployment in resource-constrained settings where access to advanced molecular testing or imaging may be limited.

Our findings also speak to current guidelines for low rectal cancer management. Both the NCCN (36) and the ESMO clinical practice guidelines (37) emphasize multidisciplinary risk stratification before neoadjuvant therapy, but neither currently incorporates a structured host immune-nutritional assessment beyond ECOG performance status and basic nutritional screening. ABINS could fill this gap with a single integer score that takes only minutes to calculate at the multidisciplinary tumor board. Importantly for clinicians, the components do not require additional blood draws beyond what is already performed routinely before neoadjuvant therapy. In our cohort, watch-and-wait management was almost five times more frequent in favorable than in poor ABINS patients (25.8% vs 5.0%), and sustained cCR rates were seven-fold higher (23.4% vs 3.3%). Although physician selection of nutritionally depleted patients away from watch-and-wait is likely to contribute to this disparity, it does not fully account for it. In a sensitivity analysis restricted to patients who underwent surgery, pathological complete response, which is an objective endpoint independent of watch-and-wait decisions, retained a strong stepwise decline across ABINS categories (26.3%, 10.5%, and 5.3%; trend P<0.001), indicating a genuine biological gradient in the probability of achieving complete response rather than physician reluctance alone. Pretreatment ABINS may therefore complement existing post-treatment criteria for watch-and-wait eligibility by providing an early signal, available before any therapy is given, of who is most likely to enter and remain in nonoperative management.

In response to reviewer comments, we have now performed a direct head-to-head comparison of ABINS with PNI, CONUT, mGPS, and CALLY in the augmented-model framing (clinical model plus each score in turn), which we regard as the clinically meaningful comparison (§3.7, **Table 9**; **Figure 5**). Adding ABINS to the clinical model yielded the highest AUC (0.768; 95% CI 0.722 to 0.810) and the highest decision-curve net benefit (0.150), and was statistically superior to clinical alone (P = 0.005) and to clinical + mGPS (P = 0.018), with a strong trend versus clinical + PNI (P = 0.051). Comparisons versus clinical + CALLY and clinical + CONUT did not reach statistical significance. In parallel single-score analyses, ABINS also ranked first for both the composite complete response endpoint (single-score AUC 0.652) and disease-free survival (c-index 0.622). These findings support ABINS as a genuinely novel instrument that captures information not fully redundant with existing scores, particularly because two of its five axes (total cholesterol and hemoglobin) are absent from CALLY, the closest single-score competitor.

The study has limitations. The retrospective design means that residual confounding by unmeasured factors cannot be fully excluded despite multivariable adjustment. We did not have data on detailed nutritional assessment such as handgrip strength, body composition by CT-derived sarcopenia metrics (30), subjective global assessment, socioeconomic status, or surveillance intensity. The sample size in the poor ABINS subgroup (n=60) and within the watch-and-wait subset (n=100) limited the precision of secondary endpoint estimates, particularly for local regrowth, where event counts in the higher-risk arms were too small for meaningful inference. In particular, only 3 patients in the poor category entered watch-and-wait management, so no valid conclusion can be drawn regarding the risk of local regrowth or the safety of nonoperative management in this high-risk subgroup (Fisher exact P = 0.595). The corresponding regrowth and sustained clinical complete response rates in the poor group should be regarded as hypothesis-generating only and must not be interpreted as evidence that watch-and-wait is either safe or unsafe for nutritionally depleted patients; this question requires dedicated prospective study in larger high-risk cohorts. ABINS uses a single pretreatment time point and does not capture longitudinal changes during therapy. Several reports suggest that on-treatment trajectories of albumin, CRP, and lymphocyte count carry incremental prognostic information beyond the baseline value (17, 23), so dynamic ABINS measurement deserves prospective evaluation. Mechanistic tissue-level data such as CD8+ T-cell infiltration, tertiary lymphoid structure density, cytokine profiles, and gut microbiome composition were not available in this cohort and could not be linked to ABINS to test mediation hypotheses (13, 32). Although our cohort spans three tertiary centers in northern China, external generalizability to other ethnic populations, healthcare systems, and immunotherapy regimens (including newer combination strategies) requires confirmation. Finally, we did not formally compare ABINS with other established host-level scores such as PNI, CONUT, modified GPS, or CALLY in head-to-head fashion. Such a comparison is a high-priority next step (17–20).

A further and important limitation, which deserves emphasis in its own right, concerns the geographic and ethnic scope of our cohort. All three participating centers are located within a single province in northern China (Hebei Province), and all patients were of Han Chinese ethnicity. This design tests within-region transportability but does not test cross-national or cross-ethnic transportability, and it does not capture healthcare systems with different neoadjuvant regimen preferences, laboratory reference ranges, or organ-preservation protocols. We therefore refrain from claims of broad generalizability, and we regard our external validation cohort as providing evidence of within-region transportability rather than global generalizability. Prospective validation in Western cohorts, in majority-MSS populations receiving contemporary total neoadjuvant therapy backbones, and in resource-constrained settings is essential before ABINS can be adopted in international practice.

In summary, ABINS is a practical, inexpensive, and biologically interpretable score for risk stratification in low rectal cancer patients receiving neoadjuvant immunotherapy. By integrating five routine pretreatment laboratory variables into a single 0 to 5 point scale, it captures host-level immune-nutritional biology that is complementary to tumor-intrinsic biomarkers and adds measurable predictive value to contemporary clinical models. Pending prospective validation, including dynamic measurement and direct comparison with other host-level scores, ABINS could become a useful component of multidisciplinary decision-making for complete response, organ preservation, toxicity management, and surveillance planning.

## Conclusions

An albumin-based immune-nutritional score (ABINS) integrating five routine pretreatment laboratory variables provides clinically meaningful risk stratification for low rectal cancer patients receiving neoadjuvant PD-1-based therapy. Higher ABINS scores were independently associated with markedly lower odds of complete response and organ preservation, substantially higher odds of clinically significant toxicity, and inferior disease-free survival. The effects were robust across temporally and geographically distinct cohorts and consistent across all 14 prespecified subgroups. Adding ABINS to the standard clinical model improved external-validation discrimination from AUC 0.703 to 0.772, with calibration slope 1.23 and intercept 0.00. Prospective validation, including dynamic on-treatment measurements and direct head-to-head comparison with existing host-level scores, is now warranted before clinical implementation.

## Data Availability

The raw data supporting the conclusions of this article will be made available by the authors, without undue reservation.

## References

[1] Sung H Ferlay J Siegel RL Laversanne M Soerjomataram I Jemal A . Global cancer statistics 2020: GLOBOCAN estimates of incidence and mortality worldwide for 36 cancers in 185 countries. CA Cancer J Clin. (2021) 71(3):209–249. doi: 10.3322/caac.21660 33538338

[2] Habr-Gama A Perez RO Nadalin W Sabbaga J Ribeiro U Silva e Sousa AH . Operative versus nonoperative treatment for stage 0 distal rectal cancer following chemoradiation therapy: long-term results. Ann Surg. (2004) 240(4):711–717. doi: 10.1097/01.sla.0000141194.27992.32 15383798 PMC1356472

[3] van der Valk MJM Hilling DE Bastiaannet E Meershoek-Klein Kranenbarg E Beets GL Figueiredo NL . Long-term outcomes of clinical complete responders after neoadjuvant treatment for rectal cancer in the International Watch & Wait Database (IWWD): an international multicentre registry study. Lancet. (2018) 391(10139):2537–2545. doi: 10.1016/S0140-6736(18)31078-X 29976470

[4] Smith JJ Strombom P Chow OS Roxburgh CS Lynn P Eaton A . Assessment of a watch-and-wait strategy for rectal cancer in patients with a complete response after neoadjuvant therapy. JAMA Oncol. (2019) 5(4):e185896. doi: 10.1001/jamaoncol.2018.5896 30629084 PMC6459120

[5] Garcia-Aguilar J Patil S Gollub MJ Kim JK Yuval JB Thompson HM . Organ preservation in patients with rectal adenocarcinoma treated with total neoadjuvant therapy. J Clin Oncol. (2022) 40(23):2546–2556. doi: 10.1200/JCO.22.00032 35483010 PMC9362876

[6] Thompson HM Omer DM Lin S Kim JK Yuval JB Verheij FS . Organ preservation and survival by clinical response grade in patients with rectal cancer treated with total neoadjuvant therapy: a secondary analysis of the OPRA randomized clinical trial. JAMA Netw Open. (2024) 7(1):e2350903. doi: 10.1001/jamanetworkopen.2023.50903 38194231 PMC10777257

[7] Cercek A Lumish M Sinopoli J Weiss J Shia J Lamendola-Essel M . PD-1 blockade in mismatch repair-deficient, locally advanced rectal cancer. N Engl J Med. (2022) 386(25):2363–2376. doi: 10.1056/NEJMoa2201445 35660797 PMC9492301

[8] Cercek A Foote MB Rousseau B Smith JJ Shia J Sinopoli J . Nonoperative management of mismatch repair-deficient tumors. N Engl J Med. (2025) 392(23):2297–2308. doi: 10.1056/NEJMoa2404512 40293177 PMC12661660

[9] Chalabi M Verschoor YL Tan PB Balduzzi S Van Lent AU Grootscholten C . Neoadjuvant immunotherapy in locally advanced mismatch repair-deficient colon cancer. N Engl J Med. (2024) 390(21):1949–1958. doi: 10.1056/NEJMoa2400634 38838311

[10] Bahadoer RR Dijkstra EA van Etten B Marijnen CAM Putter H Meershoek-Klein Kranenbarg E . Short-course radiotherapy followed by chemotherapy before total mesorectal excision (TME) versus preoperative chemoradiotherapy, TME, and optional adjuvant chemotherapy in locally advanced rectal cancer (RAPIDO): a randomised, open-label, phase 3 trial. Lancet Oncol. (2021) 22(1):29–42. doi: 10.1016/S1470-2045(20)30555-6 33301740

[11] Conroy T Bosset JF Etienne PL Rio E Francois E Mesgouez-Nebout N . Neoadjuvant chemotherapy with FOLFIRINOX and preoperative chemoradiotherapy for patients with locally advanced rectal cancer (UNICANCER-PRODIGE 23): a multicentre, randomised, open-label, phase 3 trial. Lancet Oncol. (2021) 22(5):702–715. doi: 10.1016/S1470-2045(21)00079-6 33862000

[12] Chen S Ding P Yang Y Ma S Guo H Han X . Multimodal digital biopsy for preoperative prediction of occult peritoneal metastasis in gastric cancer. NPJ Digit Med. (2026) 9(1):107. doi: 10.1038/s41746-025-02268-9 41588105 PMC12865198

[13] Hiam-Galvez KJ Allen BM Spitzer MH . Systemic immunity in cancer. Nat Rev Cancer. (2021) 21(6):345–359. doi: 10.1038/s41568-021-00347-z 33837297 PMC8034277

[14] Mantovani A Allavena P Sica A Balkwill F . Cancer-related inflammation. Nature. (2008) 454(7203):436–444. doi: 10.1038/nature07205 18650914

[15] Guven DC Sahin TK Erul E Rizzo A Ricci AD Aksoy S . The association between albumin levels and survival in patients treated with immune checkpoint inhibitors: a systematic review and meta-analysis. Front Mol Biosci. (2022) 9:1039121. doi: 10.3389/fmolb.2022.1039121 36533070 PMC9756377

[16] Ding P Yang J Guo H Wu J Wu H Li T . Multimodal artificial intelligence-based virtual biopsy for diagnosing abdominal lavage cytology-positive gastric cancer. Adv Sci (Weinh). (2025) 12(15):e2411490. doi: 10.1002/advs.202411490 39985379 PMC12005817

[17] Aoyama T Maezawa Y Hashimoto I Hara K Tamagawa A Kazama K . The CRP-albumin-lymphocyte (CALLY) index is an independent prognostic factor for gastric cancer patients who receive curative treatment. Anticancer Res. (2024) 44(4):1629–1636. doi: 10.21873/anticanres.16961 38537973

[18] Onodera T Goseki N Kosaki G . Prognostic nutritional index in gastrointestinal surgery of malnourished cancer patients. Nihon Geka Gakkai Zasshi. (1984) 85(9):1001–1005. 6438478

[19] Ignacio de Ulíbarri J González-Madroño A de Villar NGP Gonzalez P Gonzalez B Mancha A . CONUT: a tool for controlling nutritional status. First validation in a hospital population. Nutr Hosp. (2005) 20(1):38–45. 15762418

[20] Forrest LM McMillan DC McArdle CS Angerson WJ Dunlop DJ . Comparison of an inflammation-based prognostic score (GPS) with performance status (ECOG) in patients receiving platinum-based chemotherapy for inoperable non-small-cell lung cancer. Br J Cancer. (2004) 90(9):1704–1706. doi: 10.1038/sj.bjc.6601789 15150622 PMC2409737

[21] Templeton AJ McNamara MG Seruga B Vera-Badillo FE Aneja P Ocana A . Prognostic role of neutrophil-to-lymphocyte ratio in solid tumors: a systematic review and meta-analysis. J Natl Cancer Inst. (2014) 106(6):dju124. doi: 10.1093/jnci/dju124 24875653

[22] Ferrucci PF Ascierto PA Pigozzo J Del Vecchio M Maio M Antonini Cappellini GC . Baseline neutrophils and derived neutrophil-to-lymphocyte ratio: prognostic relevance in metastatic melanoma patients receiving ipilimumab. Ann Oncol. (2016) 27(4):732–738. doi: 10.1093/annonc/mdw016 26802161

[23] Ding P Chen S Guo H Yang J Fu Q Wang B . Radiomics-based ensemble model predicts postoperative recurrence of gastric cancer. BMC Med. (2025) 23(1):656. doi: 10.1186/s12916-025-04393-4 41291636 PMC12648815

[24] Ding P Yang J Chen S Guo H Wu J Wu H . Interpretable multimodal fusion model enhances postoperative recurrence prediction in gastric cancer. Adv Sci (Weinh). (2025) 12(43):e08190. doi: 10.1002/advs.202508190 40944934 PMC12631903

[25] Gu W Li H . Prognostic value of controlling nutritional status score (CONUT) in patients with colorectal cancer: a systematic review and meta-analysis. BMC Cancer. (2025) 25(1):1721. doi: 10.1186/s12885-025-15097-6 41193995 PMC12590738

[26] Pang H Dai F Zhao Z Chen X Yan M Chen L . Clinical significance of the pretreatment C-reactive protein-albumin-lymphocyte (CALLY) index in colorectal cancer patients: a meta-analysis. BMC Cancer. (2026) 26(1):212. doi: 10.1186/s12885-026-15557-7 41530698 PMC12888327

[27] Collins GS Moons KGM Dhiman P Riley RD Beam AL Van Calster B . TRIPOD+AI statement: updated guidance for reporting clinical prediction models that use regression or machine learning methods. BMJ. (2024) 385:e078378. doi: 10.1136/bmj-2023-078378 38626948 PMC11019967

[28] Dolan RD Lim J McSorley ST Horgan PG McMillan DC . The role of the systemic inflammatory response in predicting outcomes in patients with operable cancer: systematic review and meta-analysis. Sci Rep. (2017) 7:16717. doi: 10.1038/s41598-017-16955-5 29196718 PMC5711862

[29] National Cancer Institute . Common Terminology Criteria for Adverse Events (CTCAE), Version 5.0. Bethesda, MD: U.S. Department of Health and Human Services, National Institutes of Health, National Cancer Institute (2017).

[30] Prado CMM Lieffers JR McCargar LJ Reiman T Sawyer MB Martin L . Prevalence and clinical implications of sarcopenic obesity in patients with solid tumours of the respiratory and gastrointestinal tracts: a population-based study. Lancet Oncol. (2008) 9(7):629–635. doi: 10.1016/S1470-2045(08)70153-0 18539529

[31] Postow MA Sidlow R Hellmann MD . Immune-related adverse events associated with immune checkpoint blockade. N Engl J Med. (2018) 378(2):158–168. doi: 10.1056/NEJMra1703481 29320654

[32] Galon J Bruni D . Approaches to treat immune hot, altered and cold tumours with combination immunotherapies. Nat Rev Drug Discov. (2019) 18(3):197–218. doi: 10.1038/s41573-018-0007-y 30610226

[33] Wang Z Aguilar EG Luna JI Dunai C Khuat LT Le CT . Paradoxical effects of obesity on T-cell function during tumor progression and PD-1 checkpoint blockade. Nat Med. (2019) 25(1):141–151. doi: 10.1038/s41591-018-0221-5 30420753 PMC6324991

[34] Ringel AE Drijvers JM Baker GJ Catozzi A Garcia-Canaveras JC Gassaway BM . Obesity shapes metabolism in the tumor microenvironment to suppress antitumor immunity. Cell. (2020) 183(7):1848–1866.e26. doi: 10.1016/j.cell.2020.11.009 33301708 PMC8064125

[35] Ding P Chen S Guo H Yang S Wang X Han X . A deep learning-based digital biopsy for predicting early recurrence in gastric cancer. Nat Commun. (2026) 17:5220. doi: 10.1038/s41467-026-71347-6 41986314 PMC13261139

[36] Benson AB Venook AP Adam M Chang G Chen YJ Ciombor KK . NCCN guidelines insights: rectal cancer, version 3.2024. J Natl Compr Canc Netw. (2024) 22(6):366–375. doi: 10.6004/jnccn.2024.0041 39151454

[37] Glynne-Jones R Wyrwicz L Tiret E Brown G Rodel C Cervantes A . Rectal cancer: ESMO Clinical Practice Guidelines for diagnosis, treatment and follow-up. Ann Oncol. (2017) 28(suppl 4):iv22–iv40. doi: 10.1093/annonc/mdx224 28881920

